# The non-bilayer lipid MGDG stabilizes the major light-harvesting complex (LHCII) against unfolding

**DOI:** 10.1038/s41598-017-05328-7

**Published:** 2017-07-11

**Authors:** Dennis Seiwert, Hannes Witt, Andreas Janshoff, Harald Paulsen

**Affiliations:** 1 Institute of Molecular Physiology, Johannes Gutenberg University Mainz, 55128 Mainz, Germany; 2 Institute of Physical Chemistry, University of Goettingen, 37077 Göttingen, Germany

## Abstract

In the photosynthetic apparatus of plants a high proportion of LHCII protein is needed to integrate 50% non-bilayer lipid MGDG into the lamellar thylakoid membrane, but whether and how the stability of the protein is also affected is not known. Here we use single-molecule force spectroscopy to map the stability of LHCII against mechanical unfolding along the polypeptide chain as a function of oligomerization state and lipid composition. Comparing unfolding forces between monomeric and trimeric LHCII demonstrates that the stability does not increase significantly upon trimerization but can mainly be correlated with specific contact sites between adjacent monomers. In contrast, unfolding of trimeric complexes in membranes composed of different thylakoid lipids reveals that the non-bilayer lipid MGDG substantially increases the mechanical stability of LHCII in many segments of the protein compared to other lipids such as DGDG or POPG. We attribute these findings to steric matching of conically formed MGDG and the hourglass shape of trimeric LHCII, thereby extending the role of non-bilayer lipids to the structural stabilization of membrane proteins in addition to the modulation of their folding, conformation and function.

## Introduction

The major light-harvesting complex (LHCII) found in the chloroplasts of green plants contains more than half of the chlorophylls (Chl) and is the most abundant membrane protein on earth. LHCII plays a key role in photosynthesis by collecting sunlight and efficiently transferring excitation energy to the reaction centers. At the same time, LHCII serves to protect the photosynthetic apparatus from damage of excessive light and to organize the grana structures of the thylakoid membrane^1^. The 25 kDa LHCII apoprotein contains three transmembrane and two amphiphilic helices, non-covalently binding 8 Chl *a*, 6 Chl *b*, 4 carotenoid and 2 lipid molecules in a densely packed arrangement as revealed by crystal structure analysis at 2.5 Å resolution^2, 3^. LHCII monomers are assembled into trimeric complexes by the end of the greening process of the thylakoid membrane^4^, although trimerization has been proposed to be reversible upon illumination^5^. Trimerization prevents LHCII from proteolysis under high light conditions^6^ and increases its thermal stability^7^. Inspection of the LHCII crystal structure allowed to localize the contact sites between the monomers^2^, in part confirming earlier biochemical data^8, 9^. However, these findings alone provide little information about how much individual structural features contribute to the stability of the LHCII trimer or monomer.

**Figure 1:**
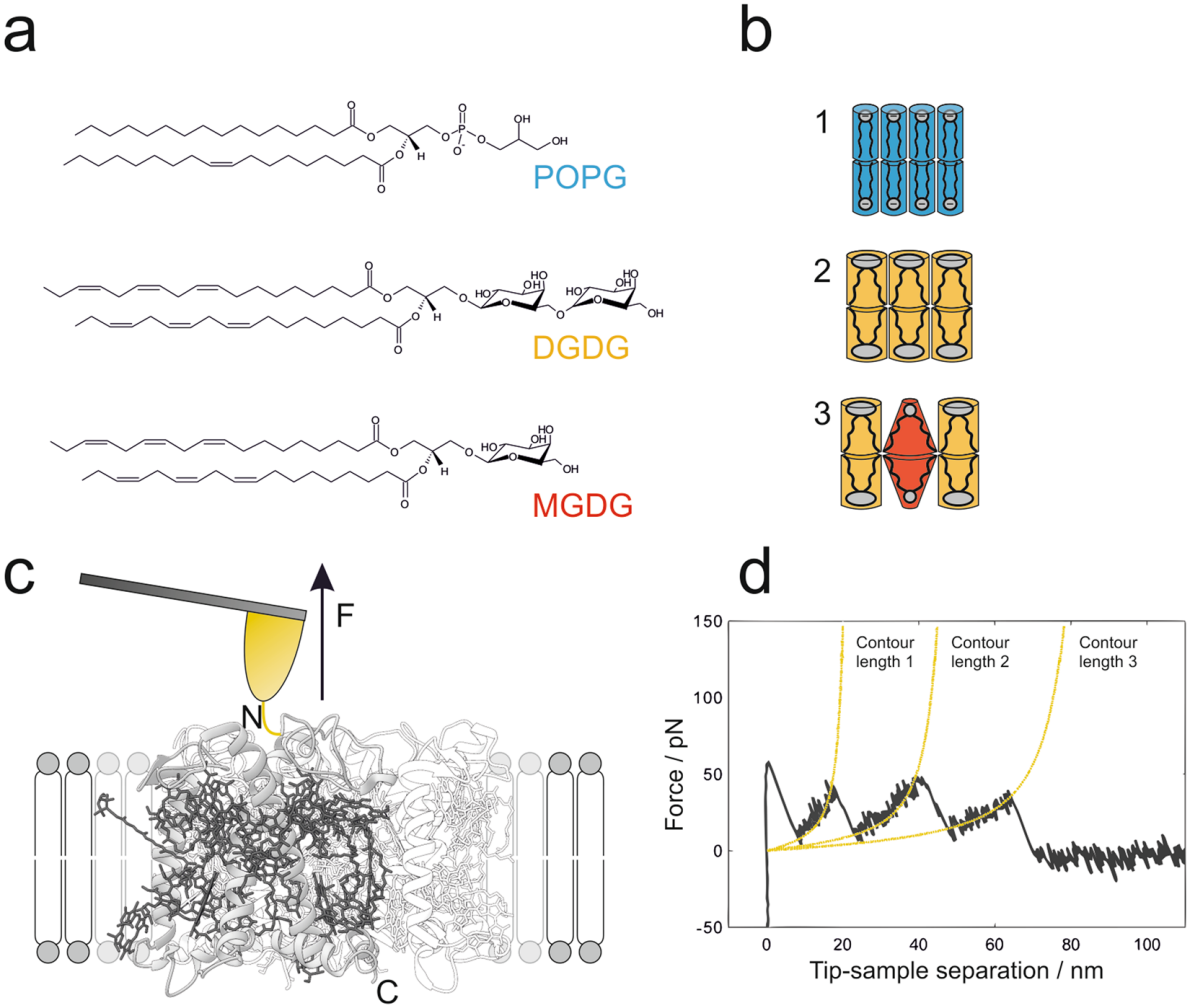
Mechanical unfolding of LHCII in membranes assembled from thylakoid lipids. (**a**) Chemical structures of model phospholipid POPG and thylakoid glycolipids DGDG and MGDG. (**b**) Lipid geometries corresponding to (**a**) and lipid compositions of the membranes applied for unfolding experiments (1–3). The degree of saturation in the alkyl chain region and the size of the head group determine the shape of lipids. Hence, POPG (blue) and DGDG (orange) adopt a cylindrical shape, forming lamellar membranes (1,2). Due to its conical shape the non-bilayer lipid MGDG (red) causes alterations in the lateral pressure profile upon incorporation into lamellar membranes^23, 24^; here: lipid ratio of DGDG / MGDG = 2:1 (3). (**c**) Schematic representation of SMFS with LHCII embedded in lipid bilayers with compositions according to (**b**). Site-directed unfolding of LHCII is achieved by covalently attaching a gold-coated AFM tip to a cysteine motif (yellow) at the amino-terminus N of the polypeptide. Retraction of the tip applies a pulling force *F* to LHCII monomers, which get extracted from the membrane out of their trimeric assembly (transparent presentation), thereby inducing stepwise unfolding of the protein; gray: polypeptide, dark grey: pigments (obtained with Chimera and PDB ID code 2BHW). (**d**) Exemplary force-distance curve recorded during the unfolding process described in (**c**). Each force peak, representing an unfolding event of LHCII, is fitted with the WLC model (yellow) which provides information about the positions of the stabilized domains (contour lengths) and the forces needed to overcome these barriers along the LHCII polypeptide.

LHCII is embedded in the thylakoid membrane, a membrane with an unusual architecture and composition. The thylakoid membrane contains 80% uncharged glycolipids (30% digalactosyl diacylglycerol (DGDG) and 50% monogalactosyl diacylglycerol (MGDG)), and 20% negatively charged lipids (10% each of phosphatidyldiacylglycerol (PG) and sulfoquinovosyl diacylglycerol (SQDG))^10^. Although the alkyl chains of DGDG are highly unsaturated (mainly 18:3, 18:3), DGDG still has a cylindrical shape due to sterical compensation by its bulky head group containing 2 galactose rings. By contrast, MGDG comprises only 1 galactose moiety and therefore adopts a conical shape with a tendency to form a hexagonal phase^11^. On the one hand, DGDG as well as the fraction of negatively charged thylakoid lipids, modelled here by 1-palmitoyl-2-oleoyl-*sn*-glycero-3-phosphoglycerol (POPG), form a lamellar phase (Fig. 1a,b). On the other hand, MGDG is a non-bilayer lipid, and even the thylakoid membrane with a MGDG fraction of 50% is unable to adopt a lamellar structure unless proteins are inserted into the membrane^12^. The role of the high content of MGDG in the thylakoid is still unclear. Proposed contributions of MGDG include structural aspects like the mediation of spontaneous curvature, the balance of excess membrane area, and specific tasks in protein functionality^13–17^. The lipid composition of chloroplasts, especially the balance of non-bilayer and bilayer lipids, is very sensitive to various kinds of external stress and a main pathway for plants to deal with changing environmental cues^18–21^.

Another intriguing feature of the thylakoid membrane is its high protein density. 70–80% of the membrane area is occupied by proteins, with LHCII comprising more than 70% of the proteins in the grana structures^22^. LHCII in particular was found to be essential for the integration of the non-bilayer lipid MGDG in high quantities into the lamellar thylakoid membrane^12^. Conversely, the presence of non-bilayer lipids in lamellar membranes is known to cause alterations in the lateral pressure profile^23, 24^, which in turn can influence the structure and function of transmembrane proteins like LHCII^25–27^. Based on energy transfer experiments in liposomes it has been hypothesized that lateral pressure exerted by MGDG facilitates the structural association of LHCII and photosystem II^28^. Spectroscopic measurements revealed that the structure of LHCII is sensitive to the molecular environment surrounding the protein^29, 30^, thus one might expect MGDG to have a notable impact on the structure and stability of LHCII. However, the presence of MGDG in liposomes has not been found to influence the thermal stability of LHCII trimers significantly^31^. In this context, new methodical approaches are required to shed light on a potential effect of lateral pressure stabilizing LHCII.

Single-molecule force spectroscopy (SMFS) is an excellent technique to study the stability of individual domains of a transmembrane protein by measuring the force needed to unfold them, while pulling them out of the membrane^32–42^. Whereas thermal denaturation experiments address the overall protein structure, SMFS provides information about subdomains by sequentially unfolding them. SMFS has also been demonstrated to be a suitable technique to characterize lipid-protein interactions^36, 37^. Recently, Scheuring and coworkers were able to apply SMFS to bacterial LH2 elucidating the free energy of oligomerization^38^. In the present work, SMFS has been used to study the mechanical stability of LHCII inserted in supported membranes as a function of lipid composition and oligomerization state. This allowed us to analyze the relationship between the structural stability of the protein and the physicochemical properties of its membrane environment, with particular emphasis on lateral pressure caused by MGDG (Fig. 1b,c). Furthermore, we investigated both trimeric and monomeric LHCII in order to localize the regions specifically contributing to trimer stability. Our findings indicate that MGDG matches the hourglass shape of LHCII thereby providing stability against unfolding.

## Results

### Mechanical unfolding of LHCII

For atomic force microscopy (AFM) based unfolding experiments, recombinant LHCII was reconstituted into preformed liposomes, which were spread on a flat mica surface. Topographical AFM images of the supported lipid bilayers revealed connected bilayer patches (Supplementary Figure 1). The glycolipids (pure DGDG or DGDG/MGDG mixtures) showed a surface coverage of approximately 30%, while POPG had an increased surface coverage of 60%. Proteins were visible as elevated objects either ~1 nm or ~1.5 nm higher than the bilayer, approximately corresponding to lumenal and stromal protrusions of LHCII, respectively^3^, with diameters on the order of 10 to 100 nm. Apparent differences in protein organization between different lipid compositions were not observed. A Cys_3_ cluster near the N-terminus of the LHCII apoprotein allowed stable and selective attachment to gold-coated AFM cantilever tips suitable for SMFS experiments (Fig. 1c). Insertion of recombinant LHCII in artificial membranes presumably results in random up-down orientation; however, the N-proximal Cys_3_ attachment site permits to select those complexes for unfolding that expose their stromal surface^32^. The cantilever tip was brought into contact with the membrane surface and was then retracted to unfold the peptide chain as indicated by the saw tooth signature, characteristic for protein unfolding (Fig. 1d), with each force peak representing unfolding of an individual segment of the polypeptide chain^34, 35^. Only force-distance curves showing unfolding of the complete peptide chain were taken into account for further analysis as indicated by a force peak in a 10 nm interval around the contour length of the LHCII monomer estimated to be 88 nm. Each individual unfolding peak was fitted using the worm-like chain (WLC) model assuming a persistence length of 0.4 nm^43, 44^ to assess the contour length *l*_*C*_ of the unfolded segment and the corresponding unfolding forces (Fig. 1d).

**Figure 2:**
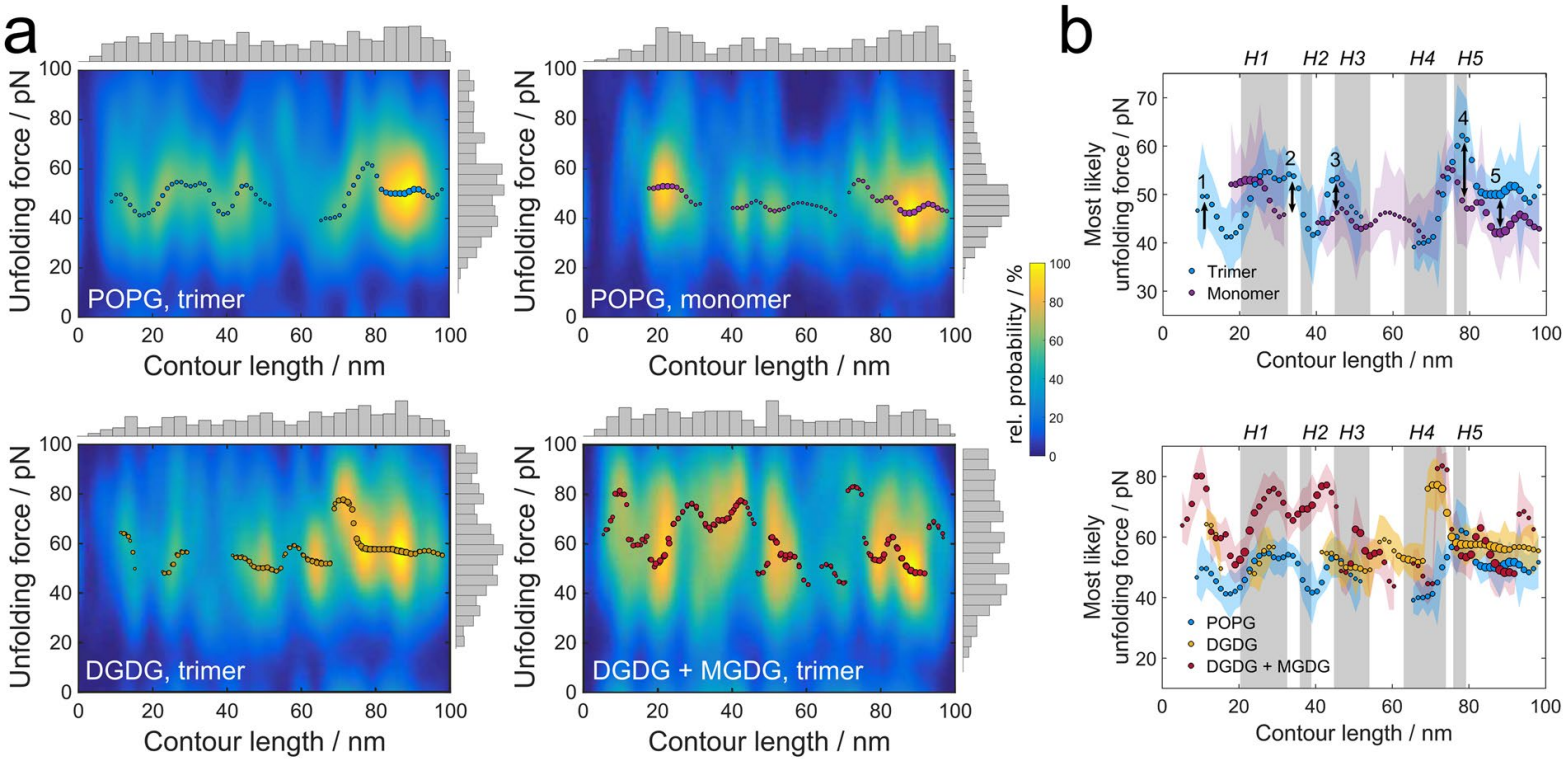
Unfolding forces of LHCII depend on trimerization and the lipid matrix. (**a**) Multivariate kernel density estimate of the unfolding force as a function of the contour length for LHCII monomers in POPG (top right) and LHCII trimers in POPG (top left), pure DGDG (bottom left) and a mixture of DGDG and MGDG (bottom right). The dots mark the most likely unfolding forces for a given contour length, while the diameter of the dots represents their relative probability of at least 40% in relation to the most probable unfolding event. The histograms (grey) at the axes show the individual underlying distributions of unfolding forces and contour lengths. (**b**) Most likely unfolding force as a function of the contour length (obtained from the dots in (**a**)) as a function of oligomerization state (top) and lipid mixture (bottom). Helices (H1-5) are indicated as shaded gray areas. The color shaded areas indicate the standard error of the most likely unfolding force. The trimer shows enhanced stabilization compared to the monomer in distinct domains of the protein, indicated by numbers 1 to 5. Both glycolipids provide additional stability for trimeric LHCII at helix 4, while the addition of MGDG strongly increases the unfolding forces in many segments along the LHCII polypeptide.

Frequent observation of a particular unfolding peak is usually linked to stable structural elements of the mechanically challenged protein that need not necessarily be identical with secondary-structure components^33^. Interestingly, the histogram of all contour lengths obtained by the WLC fits (Fig. 2a, shown as histograms on the x-axes) revealed a very broad distribution with unfolding events almost evenly spread over the full length of the peptide chain. In contrast to unfolding experiments on numerous other proteins distinct peaks were absent (as illustrated in Supplementary Figure 2)^32, 36, 37, 39–42^. The broad distribution might be attributed to the complex structure of LHCII forming a superhelix in combination with a high number of bound auxiliary pigments (see Fig. 1c)^2, 3, 45^. In contrast to the contour length histograms, the corresponding unfolding force histograms are less broad and show a clear preference for unfolding forces around 50 pN dependent on the lipid matrix and the oligomerization state (Fig. 2a, shown as histograms on the y-axes).

The common procedure for analysis of unfolding force curves is to map recurring unfolding peaks to stable elements and categorize every detected unfolding event accordingly. Due to the broad distribution of detected contour lengths such an analysis would be very unreliable and prone to false assignment of peaks to structural elements. Therefore, in order to circumvent categorization of unfolding events to structural elements, we estimated the probability to find a certain unfolding force at a given contour length as obtained from the WLC fits by using a multivariate kernel density estimate (Fig. 2a). Analysis of this “stability map” then allowed us to relate the mechanical response of the polypeptide to the stabilization of structural elements depending on different intrinsic and environmental conditions (see below). In order to compare unfolding forces between different experiments we considered the most likely unfolding force as a function of the contour length (colored dots in Fig. 2a,b). To find the most prominent contributions to protein stability, only unfolding events with a likelihood of at least 40% of the most probable unfolding event were selected. This also sorts out unfolding events with unfolding forces above 100 pN, which we attribute to simultaneous unfolding of more than one monomer attached to the cantilever in parallel.

### Impact of LHCII trimerization

LHCII monomers inserted in PG-containing membranes tend to trimerize spontaneously^46^; therefore, in order to address the impact of trimerization on the mechanical stability of LHCII, a LHCII variant was used with two point mutations in its trimerization motif (positions 16 and 17) that render the protein incapable of trimerization, as described earlier^8^. For comparing LHCII monomers and trimers, the complexes were inserted into membranes consisting of POPG exclusively. The distribution of unfolding forces (Fig. 2a, shown as histograms on the y-axes) reveals that the most frequent unfolding force is increased by around 10 pN from monomers to trimers, demonstrating a slightly stabilizing effect of trimerization on LHCII. This is in agreement with previous thermal denaturation experiments revealing an increased stability of the trimer^7^.

Comparing the most likely unfolding force at each contour length (colored dots in Fig. 2a top left, top right) for monomeric LHCII and a single LHCII polypeptide unfolded out of the trimer reveals selective stabilization in the trimer. In some contour length intervals unfolding forces in the trimer are similar to those in the monomer, whereas in other parts of the polypeptide -although most of the disparities are not significant unless stated otherwise- the forces are higher for trimers than for monomers (Fig. 2b top). While the N-terminal hydrophilic domain at contour lengths of *l*_*C*_ ≈ 10 nm shows no detectable unfolding events for the monomer, there is a high probability to observe unfolding events for trimeric LHCII (Fig. 2b top, indicated by 1). Along those lines, unfolding forces of the trimer were generally higher from *l*_*C*_ ≈ 27 nm to *l*_*C*_ ≈ 34 nm (*∆F* ≈ 8 pN), at *l*_*C*_ ≈ 47 nm (*∆F* ≈ 7 pN), *l*_*C*_ ≈ 77 nm (*∆F* ≈ 12 pN), and for the C-terminus at *l*_*C*_ > 82 nm (*∆F* ≈ 8 pN, same figure, indicated by 2, 3, 4 and 5, respectively). Interestingly, in two segments of the protein, unfolding forces were slightly higher for the monomer than for the trimer (from *l*_*C*_ ≈ 15 nm to *l*_*C*_ ≈ 25 nm and from *l*_*C*_ ≈ 65 nm to *l*_*C*_ ≈ 70 nm).

Stabilization of the trimer compared to the monomer, as indicated by higher unfolding forces, can be linked to interactions in distinct protein domains facilitating LHCII trimerization. Both the N-terminus and the C-terminus have previously been described to be important for trimerization based on mutation analysis^8, 9^ and structural data^2^, which is mirrored here in the significant stabilization at *l*_*C*_ ≈ 10 nm (peak 1) and the stabilization at *l*_*C*_ > 82 nm (peak 5). The key trimerization motif has been identified as WYGPDR at aa17–aa22 (*l*_*C*_ = 6–8 nm)^8^. However, detaching the cantilever tip from the membrane surface causes adhesion peaks at the beginning of each force-distance curve that may obscure unfolding events in the very proximal segment of the N-terminus. Hence, the precision in determining contour lengths in this domain is not sufficient to be sure that the peak at *l*_*C*_ ≈ 10 nm is in fact due to the trimerization motif.

A critical factor for trimerization is the binding of pigments and lipids. Liu *et al*. pointed out an important role of helix 3 by coordinating several Chl *b* molecules, mediating hydrophobic interactions between adjacent monomers^2^. Here, we may observe a contribution to trimer stability by one chlorophyll in particular: Chl8 (nomenclature according to Standfuß *et al*.^3^) is bound to two different monomers via coordination of the central Mg^2+^ by His212 (*l*_*C*_ = 77 nm, corresponding to peak 4 within helix 5) of one monomer and hydrophobic interaction of the phytol tail with Trp128 (*l*_*C*_ = 47 nm, corresponding to peak 3 at the beginning of helix 3) of the other monomer (Supplementary Figure 3). In addition, Trp222 (*l*_*C*_ = 81 nm) has been described to be a sensitive site for trimer formation and stability^9, 45^, which may explain an enhanced stabilizing effect at peak 4 compared to the other regions.

Liu *et al*. identified Chl4 and Chl5 bound via Glu65 (*l*_*C*_ = 24 nm) and His68 (*l*_*C*_ = 25 nm), respectively, to be crucial for the trimer structure^2^. However, unfolding forces of trimeric and monomeric LHCII show little differences at these contour lengths. Strong intramolecular interactions, like the salt bridge at the helix cross between helix 1 at Arg70 (*l*_*C*_ = 25 nm) and helix 3^45^, may be dominating in this segment, obscuring the effect of neighboring monomers as discussed by Sapra *et al*. for bacteriorhodopsin assemblies^47^.

There is no straightforward assignment of the significant stabilization between *l*_*C*_ ≈ 27 nm and *l*_*C*_ ≈ 34 nm (region 2) to the structure of LHCII. This contour length interval corresponds to the lumenal end of the transmembrane helix 1 and the amphiphilic helix 2. However, Liu *et al*. found a highly reduced degree of trimerization after an exchange of W97 to alanine (*l*_*C*_ = 35 nm) at the beginning of helix 2^48^, representing a binding site for lutein 2^45^. This pigment has been suggested to assume a trimer specific conformation^7^, indicating that lutein 2 and hence W97 might contribute to the stability of trimeric LHCII to a certain extent as reflected by our data.

It seems surprising that we observed one position at the N-terminal domain close to helix 1 and one at the beginning of helix 4 with higher unfolding forces for the monomer than for the trimer. The PG molecule bound to LHCII via Tyr44 (*l*_*C*_ = 16 nm) and Lys182 (*l*_*C*_ = 66 nm)^3^ has been identified to be a major factor in trimerization^49, 50^. Moreover, there are indications that the monomeric complex alone is not able to bind PG^50^. While PG facilitates trimer formation, electrostatic repulsion between the negatively charged head groups of the bound lipid and the membrane lipids is energetically unfavorable and might destabilize the trimeric protein at these positions in a POPG matrix.

### Effect of lipid matrix

To assess the impact of the lipid matrix on the mechanical stability of LHCII we performed unfolding experiments with trimeric LHCII in three different lipid matrices: pure POPG, pure DGDG and a mixture of DGDG and MGDG (2:1). Lipid mixtures containing more than roughly 30% MGDG no longer form lipid vesicles^51^, therefore we abstained from using higher percentages of MGDG, expecting that if the lateral membrane pressure has an effect on LHCII, this is seen at 30% MGDG. Since neither of the bilayer lipids POPG and DGDG affects the lateral pressure profile of a lamellar membrane, differences in unfolding forces between these lipid environments can primarily be attributed to the charge of the lipid head groups (Fig. 1a,b). Alterations in protein stability upon addition of 30% non-bilayer lipid MGDG to DGDG membranes provide information about the impact of the modified lateral pressure profile^24^, while the charge of the membrane and the chemical properties of the head groups remain unaffected.

The distribution of unfolding forces varies only little between the two cylindrical lipids POPG and DGDG (Fig. 2a top left, bottom left; shown on the y-axes). When considering the most likely unfolding force for every contour length, the N-terminus around *l*_*C*_ ≈ 10 nm and the region from the end of the stromal loop to the beginning of helix 4 around *l*_*C*_ ≈ 60 nm are slightly more stable (*∆F* ≈ 10 pN) in DGDG compared to POPG (Fig. 2b bottom). The lumenal side of helix 4 from *l*_*C*_ ≈ 70 nm to *l*_*C*_ ≈ 75 nm is greatly stabilized (*∆F* ≈ 25 pN) in both glycolipid matrices (DGDG and DGDG + MGDG). This narrow peak points to a protein domain interacting with lipid head groups on the lumenal side of helix 4.

Adding MGDG to the DGDG membrane significantly raises the overall occurrence of unfolding forces above 70 pN (Fig. 2a bottom left, bottom right; shown on the y-axes) and strongly enhances the unfolding forces in several segments of the protein (Fig. 2b bottom). Unfolding forces are increased by up to 20 pN from *l*_*C*_ ≈ 6 nm to *l*_*C*_ ≈ 12 nm close to the N-terminus, from *l*_*C*_ ≈ 24 nm to *l*_*C*_ ≈ 32 nm within helix 1, from *l*_*C*_ ≈ 34 nm to *l*_*C*_ ≈ 46 nm comprising helix 2, the lumenal loop and part of helix 3, and from *l*_*C*_ ≈ 50 nm to *l*_*C*_ ≈ 54 nm at the stromal side of helix 3. In contrast to the protein stabilization due to trimer formation, the stabilized regions along the peptide chain due to the surrounding lipids are rather broad extending over up to 30 amino acids, indicating a regional stabilization rather than local interactions between individual amino acids.

## Discussion

### Unfolding monomers and trimers reveals a connection between structure and stability of LHCII, despite the structural intricacy of the pigment-protein complex

Site-directed and complete unfolding of recombinant LHCII by SMFS was facilitated by linking the cantilever to an N-proximally introduced Cys_3_ cluster^32^. In contrast to other transmembrane proteins studied by SMFS^32, 36, 37, 39–42^, unfolding of different LHCII peptides does not always follow the same unfolding trajectory as indicated by the notable absence of clear peaks in the unfolding contour length histograms (Fig. 2a; see also Supplementary Figure 2). Since the broad distribution of contour lengths could in principle also arise from careless data evaluation or experimental errors, a number of quality control measures were taken to minimize the impact of those effects. To this end, the protein was site-specifically linked to the cantilever at the amino-terminus, with the aim to reduce the impact of nonspecific adhesion and random pulling from the stromal or the lumenal side during the unfolding experiments. Notably we do not observe any symmetry in the most frequent unfolding forces along the LHCII polypeptide, which would indicate stochastic pulling from either the N- or the C-terminus (Fig. 2b). Nonspecific attachment of the cantilever to the stromal or lumenal loop leads to force-distance curves with unfolding events at tip-sample separations much shorter than 78 nm, which were excluded from analysis. Similarly, force-distance curves lacking the characteristic WLC shape of protein unfolding were not analyzed. Additionally, the AFM setup used here has low instrumental noise with a standard deviation of 4 pN due to rigorous screening against external acoustic sources.

Therefore, we ascribe our findings to the intricate structure of the protein. The two tilted helices 1 and 4 are interlocked by two salt bridges, forming a super-secondary structure^3, 45^ (see Fig. 1c). In addition, the high number of pigments coordinated by LHCII (18 per monomer) leads to added links between different segments of the polypeptide, each capable to stabilize the protein structure. Considering more than 20 known pigment binding sites^45^ and the contour length of the LHCII monomer of 88 nm, the average distance along the peptide chain between two pigment binding sites is around 4 nm. Assuming an experimental error for the determination of the contour length of an unfolded segment of 2 nm (as a lower bound), it becomes clear that adjacent pigment binding sites cannot be resolved with the current experimental capabilities according to this simple calculation.

By analyzing the most likely unfolding force at every position along the polypeptide chain we avoided to focus on selected structural elements in the protein originating from the frequency of contour lengths only. We used this approach to analyze unfolding curves of the LHCII trimer and an LHCII point mutant incapable of trimerization, revealing localized differences in unfolding forces between monomeric and trimeric LHCII which can be directly linked to the protein structure. Although not at all positions along the contour of the protein statistically secured, we find this approach for data analysis reasonable as the disparities in unfolding forces due to trimerization can be largely correlated with previously published structural and biochemical data. We therefore relied on the method to further explore how the stability of LHCII is modulated by the lipid matrix surrounding the protein. In contrast to the rather subtle impact of oligomerization on the unfolding forces of LHCII we found that the lipid matrix has a substantial influence on protein stability as discussed below.

### Lateral pressure by MGDG greatly stabilizes trimeric LHCII due to its hourglass shape

The regions of the LHCII trimer that are stabilized in DGDG compared to POPG membranes are mainly located in the extra-membrane domains of the protein in plane with the lipid head groups, suggesting a destabilizing impact of the negatively charged phosphate group and/or stabilizing effects by the galactose moieties (Fig. 2b bottom). The N-terminal region from *l*_*C*_ ≈ 11 nm to *l*_*C*_ ≈ 13 nm is rich in polar amino acids (Ser29, Ser32, Ser34, Tyr35 and Thr37) capable of forming hydrogen bonds with the sugar residues, which may lead to an increased stability by both glycolipids (MGDG and DGDG) in this segment of the polypeptide. Negatively charged amino acids (Asp162, Asp168, Asp169 and Glu171) in the stromal loop from *l*_*C*_ ≈ 59 nm to *l*_*C*_ ≈ 62 nm may generate repulsive ionic interactions with phosphate head groups and thus destabilize the protein at the end of the stromal hydrophilic domain in a POPG matrix. In addition, repulsive forces due to the bound PG-molecule at *l*_*C*_ ≈ 66 nm may extend the destabilization of this segment to the stromal side of helix 4, similar to the statement made above for the comparison of the monomer and the trimer^3^. The greatly enhanced stabilization of LHCII against unfolding by both glycolipid mixtures at the lumenal side of helix 4 around *l*_*C*_ ≈ 75 nm probably arises from hydrogen bonds between the corresponding polar amino acids (Gln197, Thr201 and Lys203) and the galactose rings of either DGDG or MGDG, as the presence of positively charged Lys203 renders a repulsive ionic interaction with the POPG head group highly unlikely. However, besides the vast chemical differences between POPG and DGDG, especially considering the different charges, disparities in unfolding force are minuscule.

**Figure 3:**
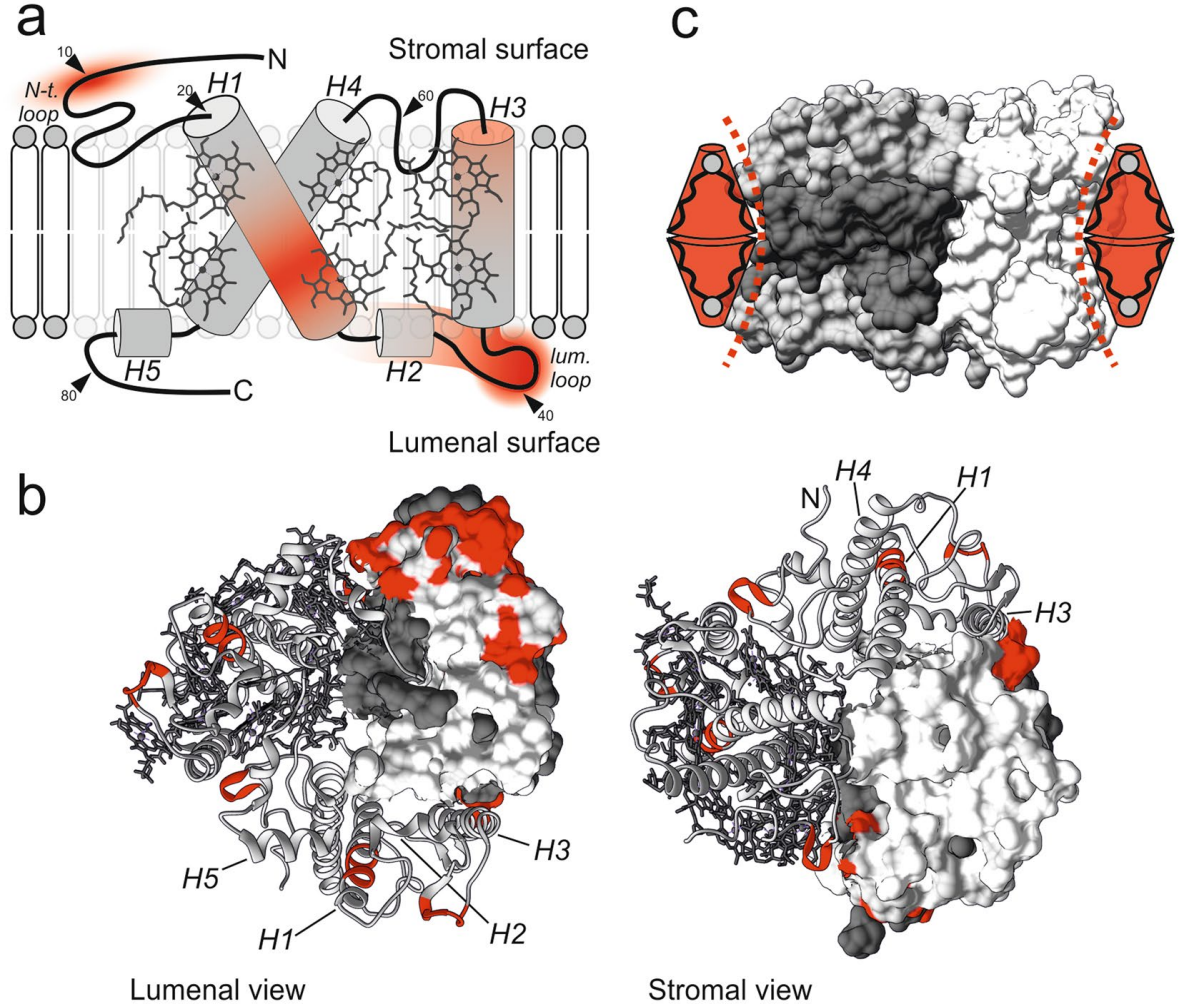
Lateral pressure by MGDG stabilizes LHCII in a structure dependent manner. (**a**) Schematic presentation of the LHCII monomer structure with degree of stabilization by MGDG (red color gradient) derived from Fig. 2b. Stabilization comprises domains in the transmembrane (helices H1 and H3) and extra-membrane domains (N-terminus, H2 and lumenal loop) of the protein; small arrows indicate contour length positions along the LHCII polypeptide. (**b**) Hotspots of MGDG mediated stabilization (red) in the crystal structure of LHCII from lumenal and stromal view corresponding to the highest intensities in (**a**) and *l*_C_ ± 1 nm of the peak centers in Fig. 2b respectively. The effect of MGDG is the strongest in the periphery of the protein. Within a trimer, the monomers are represented by the surface structure, the tertiary structure (helices H1-H5 indicated) with and without pigments; gray or white: polypeptide, dark gray: pigments. (**c**) Steric interplay between trimeric LHCII and MGDG. Stabilization of LHCII is accomplished as the concave-like surface structure of trimeric LHCII (emphasized with dashed lines) matches the conical shape of MGDG (red); colors (gray: polypeptide, dark gray: pigments) highlight one LHCII monomer within the trimeric assembly. Structures obtained with Chimera and PDB ID code 2BHW.

By contrast, large differences are seen in unfolding forces between the DGDG/MGDG mixture and pure DGDG, despite the similarities between DGDG and MGDG with regard to charge and functional groups. This rules out specific or electrostatic interactions as a reason for the mechanical stabilization. The stabilizing effect of MGDG is not restricted across the membrane plane since it includes transmembrane as well as extra-membrane domains of LHCII (shown as schematic representation in Fig. 3a). A view on the stromal and lumenal faces of LHCII reveals that the stabilization is confined to the periphery of the trimer where it is in direct contact with the lipid matrix (Fig. 3b). We therefore attribute the stabilization to steric interactions between the hourglass shape of trimeric LHCII and the conical shape of MGDG (Fig. 3c), pointing to bulk physical properties of the lipid since it is not part of the LHCII structure^2^. Our results are in accordance with recent simulations demonstrating that concavely formed proteins are energetically favored in membrane domains exhibiting negative curvature stress^52^, which is a measure for alterations in the lateral membrane pressure profile induced by non-bilayer lipids such as MGDG^24, 26^. Yang *et al*. monitored the dissociation of LHCII trimers into monomers as a function of thylakoid lipid composition, showing only a slight effect of MGDG^31^. This is in agreement with our data as the impact of MGDG is particularly intense in the periphery of LHCII (Fig. 3b), whereas trimerization is mainly based on pigment mediated interactions in the hydrophobic core^2^.

A lot of attention has been paid to how non-bilayer lipids, particularly phosphatidylethanolamine (PE) representing up to 38% of the inner mitochondrial membrane lipids^53^, modulate the folding, conformation and functionality of integral membrane proteins^26, 27^ (see also references therein). However, only little is known about their impact on the stability of transmembrane proteins, even though complementary shapes of transmembrane proteins and lipids appear to be a widespread phenomenon. The concavely formed ion channel gramicidin and the non-bilayer lipid lysophosphatidylcholine as well as the potassium channel KcsA and PE are prominent examples for the proposed steric interplay^54, 55^. Unfolding of LHCII via SMFS allowed us to observe a stabilizing effect of complementary lipid and protein shapes (as illustrated in Fig. 3c), thereby displaying the impact of the lateral pressure profile over the whole membrane cross section (Fig. 3a). While LHCII is needed to stabilize MGDG in a lamellar phase, our data clearly show that vice versa MGDG increases the mechanical stability of LHCII. Our results will contribute to the ongoing discussion of the role on non-bilayer lipids in many biological membranes.

## Methods

### Preparation of recombinant LHCII

The LHCII variants used in this study were derivatives of Lhcb1*2 (AB80) gene from pea (*Pisum sativum*)^56^ containing a C-terminal hexahistidine (His_6_) tag and having the native cysteine at position 79 replaced with serine. In order to achieve site-specific unfolding a cysteine motif (Cys-Cys-Cys) was added at the N-terminus of the protein by using the Phusion site-directed mutagenesis kit (Thermo Scientific). This version also served as a template to construct the LHCII variant WY16,17AV^8^ by using the QuikChange Lightning site-directed mutagenesis kit (Agilent Technologies), which was applied for the monomer studies. The genes were inserted in the pDS12-RBSII vector and overexpressed overnight in *Escherichia coli* strain JM101, followed by isolation of the apoprotein according to established protocols^57^. The apoprotein was reconstituted to monomeric LHCII with total pigment extract from pea thylakoids^58^ by the detergent exchange method^59^. His_6_ tag mediated trimerization of the monomers was performed on Ni^2+^-sepharose columns^60^, followed by purification via ultracentrifugation through a sucrose density gradient as described earlier^61^, but using a different buffer (500 mM sucrose, 10 mM Tris-HCl pH 7.5, 5 mM TCEP, 0.05% (w/v) Triton X-100). In case of the variant WY16,17AV the trimerization step was omitted and the reconstitution mix was directly loaded on the sucrose gradient containing a higher concentration of Triton X-100 (0.1%). The bands corresponding to LHCII trimers or monomers (in case of WY16,17AV) respectively were collected and checked for correct assembly via CD spectroscopy on a J-810 spectropolarimeter (Jasco) in the visible range, with trimeric LHCII exhibiting a characteristic negative peak at 474 nm^61^. For insertion into preformed liposomes the buffer was exchanged to AFM buffer (50 mM NaCl, 10 mM Tris-HCl pH 7.5, 5 mM TCEP) using Amicon 30-kDa centrifugal filters, while the suspension still contained Triton X-100.

### Reconstitution of LHCII into liposomes

Lipid aliquots of pure POPG (purchased from Avanti Polar Lipids), pure DGDG or a mixture of DGDG and MGDG (each lipid purchased from Lipid Products) at a molar ratio of 2:1 were dissolved in chloroform. The chloroform was slowly removed in a rotary evaporator by which a thin lipid film was formed on the inner wall of a glass flask. After complete removal of the solvent under high vacuum at 40 °C for 1 h, the lipid film was hydrated by rigorous mixing (3 × 30 s at 60 °C, MS2 minishaker, IKA) in AFM buffer at a total lipid concentration of 1.5 mM to produce preformed liposomes. For MGDG containing samples the mixing step was repeated several times until no more lipid traces could be detected by eye on the bottom of the flask. Sonication in a tip sonicator (Vibra cell, Sonics & Materials) for 4 min, followed by 3 freeze-thaw cycles, yielded large unilamellar vesicles. The vesicle suspension was then extruded 21 times (LipoFast-Basic, Avestin) through a polycarbonate membrane (pore diameter 100 nm) and mixed with Triton X-100 to a final concentration of 0.05% (w/v). Reconstitution of recombinant LHCII into preformed liposomes was performed according to established protocols^31^, but with some modifications: the protein suspension was added dropwise under continuous mixing at 4 °C in the dark to a molar lipid/protein ratio of 500–1000, and subsequently polystyrene beads (Bio-Beads SM-2; Bio-RAD) at 30 mg/ml were added. In order to remove Triton X-100 the mixture was incubated overnight under constant rotating at 4 °C in the dark, and the supernatant was removed to a new tube containing fresh Bio-Beads, followed by incubation for 1 h. The last step was repeated, and finally the supernatant was collected. The inserted LHCII trimers or monomers were checked for integrity on a FluoroMax-2 spectrometer (Horiba Scientific) as described earlier^60^.

### SMFS experiments

Prior to the SMFS experiments, proteoliposomes were extruded 21 times (LipoFast-Basic, Avestin) through a polycarbonate membrane (pore diameter 100 nm). CaCl_2_ solution (100 mM) was added to a final concentration of 5 mM and the suspension (60 µl) was incubated for 10 min on freshly cleaved mica, leading to surface supported bilayers containing LHCII proteins (Supplementary Figure 1). Nonadsorbed vesicles were removed by intensive rinsing (4 × 1 ml) with AFM buffer. SMFS measurements were conducted in AFM buffer at room temperature using a commercial atomic force microscope (MFP-3D Infinity, Oxford Instruments Asylum Research) and gold-coated silicon nitride cantilevers (Biolever, Olympus). Each cantilever was calibrated with the thermal noise method yielding spring constants around 6 pN/nm. The cantilever was moved toward the surface at 500 nm/s until a load force of 100 pN was reached. The tip was kept in contact with the membrane for 1 s to allow for binding of the Cys_3_-motif at the N-terminus of LHCII to the gold-coated cantilever tip. The cantilever was then retracted at 1000 nm/s to unfold the peptide, giving rise to a force-distance curve. For each sample only a single force-distance curve was collected within an area of 1 µm^2^ and each area of the supported bilayer was only scanned once. Roughly 10% of the curves showed force peaks corresponding to unfolding events of the protein, of which ~7% exhibited a force peak corresponding to a contour length of 88 ± 10 nm indicating full unfolding of the LHCII polypeptide. Only the latter were selected for further analysis, thus more than 25,000 force-distance curves were recorded for each experimental condition to obtain a sufficient number of curves showing full unfolding (monomer in POPG: *n = *193 out of ~30,000 curves; trimer in POPG: *n = *328 out of ~50,000 curves; trimer in DGDG: *n = *262 out of ~50,000 curves; trimer in DGDG/MGDG: *n = *195 out of ~25,000 curves). The SMFS measurements were repeated at least 3 times for each experimental condition.

### SMFS data analysis

Only force-distance curves showing the saw tooth signature characteristic for protein unfolding with an unfolding peak corresponding to a contour length of 88 ± 10 nm were selected for further analysis. Each individual unfolding peak was fitted using a WLC model^43, 44^:

$$F(x)=\,\frac{{k}_{B}T}{b}(\frac{1}{4}{(1-\frac{x}{{l}_{C}})}^{-2}-\frac{1}{4}+\frac{x}{{l}_{C}})$$

with the force $F(x)\,$ at tip-sample separation $\,x$, the Boltzmann constant ${k}_{B}$, the persistence length $b=4\,\AA$, the contour length of the unfolded peptide ${l}_{C}\,$ and the temperature $T=298\,\text{K}$. Additionally, the peak force ${F}_{max}$ of each unfolding peak was recorded. To estimate the distribution of the value pairs of ${l}_{C}\,$ and ${F}_{max}$ for all recorded unfolding events, we calculated a multivariate kernel density estimate using the approximated experimental uncertainty $\sigma ({l}_{C})=2\,\mathrm{nm}$ and $\sigma (F)=12\,\mathrm{pN}$ as kernel bandwidths, as estimated from the standard deviation of the measured force during the baseline of the force-distance curve, i.e. recorded far away from the surface. The uncertainty of the contour length was calculated from the uncertainty of the measured force and the spring constant. This gives us an unfolding force distribution at any given contour length. The maxima of these distributions give us the most likely unfolding force ${F}^{\ast }$ as a function of the contour length. The most likely unfolding force was evaluated at discrete contour lengths with a fixed distance between two points Δ*l*_*C*_ = 1.25 nm. As an estimate of the uncertainty of the most likely unfolding force we calculated the standard error of the most likely unfolding force $\sigma ({F}^{\ast })$ for each contour length ${l}_{C}\,$ as $\sigma ({F}^{\ast })=\sqrt{\frac{{\rm{Var}}({F}_{max})}{N}}$, using the variance ${\rm{Var}}({F}_{max})$ of all measured unfolding forces ${F}_{max}$ in the interval ${l}_{C}\pm \frac{1}{2}{\rm{\Delta }}{l}_{C}$ and the number of data points in that interval $N$.

### Structure representation

The LHCII structure (PDB ID 2BHW)^3^ representation was created using the UCSF chimera package^62^.

### Data Availability

The datasets generated during and/or analyzed during the current study are available from the corresponding author on reasonable request.

## Electronic supplementary material

Supplementary Information
