# Supplementary material for: The non-bilayer lipid MGDG stabilizes the major light-harvesting complex (LHCII) against unfolding
Source: Sci Rep. 2017 Jul 11;7:5158. doi: 10.1038/s41598-017-05328-7 (PMC5505961; doi:10.1038/s41598-017-05328-7)
Supplement: Supplementary file 1 — Supplementary Information [file 41598_2017_5328_MOESM1_ESM.pdf]

# Supplementary information

# The non-bilayer lipid MGDG stabilizes the major light-harvesting complex (LHCII) against unfolding

Dennis Seiwert[a,1], Hannes Witt[b,1], Andreas Janshoff[b,2], Harald Paulsen[a,2]

[a] Institute of Molecular Physiology, Johannes Gutenberg University Mainz, Germany;
[b] Institute of Physical Chemistry, Georg-August University, Göttingen, Germany

[1] Co-first author

[2] Co-corresponding author: ajansho@gwdg.de, paulsen@uni-mainz.de

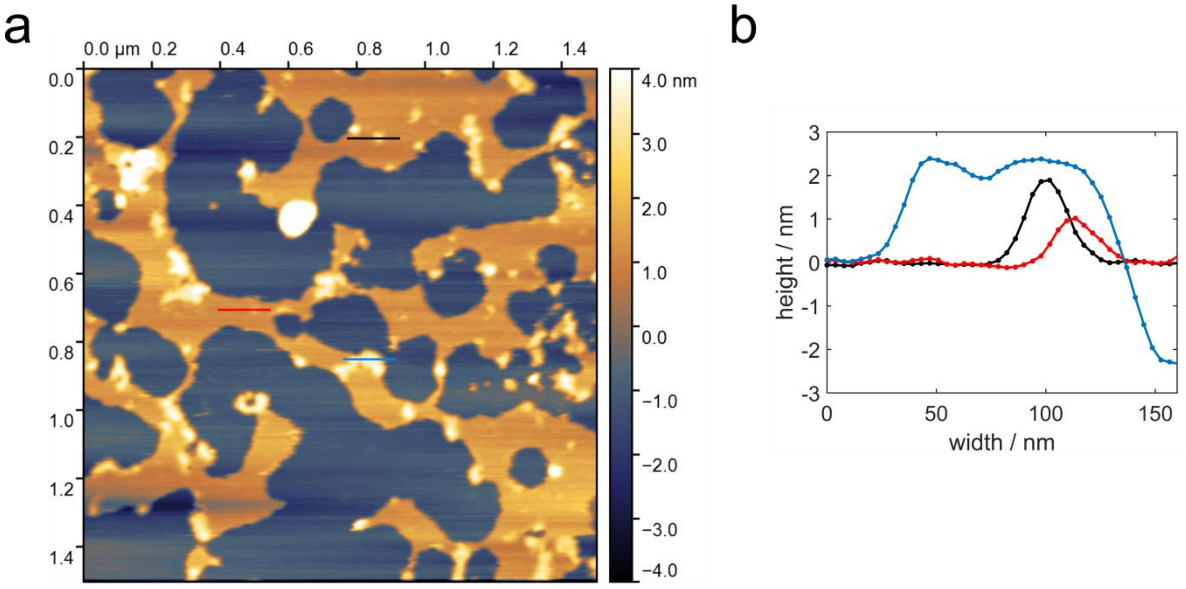


**Supplementary Figure 1** Topography of trimeric LHCII reconstituted into DGDG membranes as obtained from AFM imaging. (**a**) Incubation of LHCII containing liposomes on mica leads to the formation of connected bilayer patches, appearing as protrusions from the mica surface (dark, bluish color) between 2 and 3 nm in height (bright, yellowish color). The deviation from the expected bilayer thickness of 3 to 4 nm can be explained by compression of the soft bilayer due to the force applied by the AFM during imaging. (**b**) Height profile analysis of selected features within the supported bilayers. Deposited membrane patches, around 2 nm in height (black, blue), can be distinguished from smaller patches corresponding to proteins protruding 1 to 1.5 nm from the bilayer surface (red). Imaging was performed in intermittent contact mode.

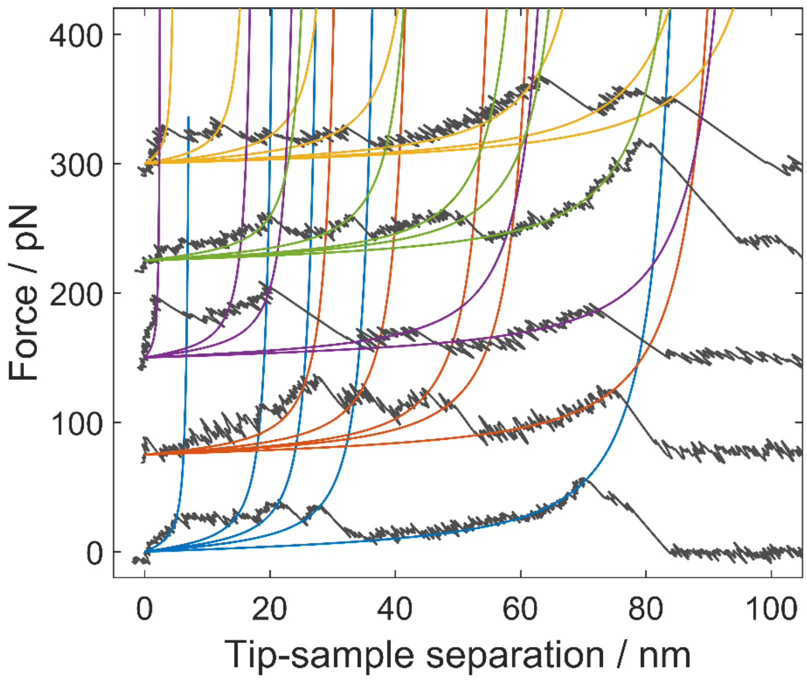


**Supplementary Figure 2** Exemplary force-distance curves showing mechanical unfolding of LHCII polypeptides extracted from the trimeric LHCII in POPG. Each force peak is fitted with the WLC model shown as colored lines. Curves are offset for better visibility. Notably each curve shows a different saw tooth pattern indicating that unfolding of the protein follows different trajectories.

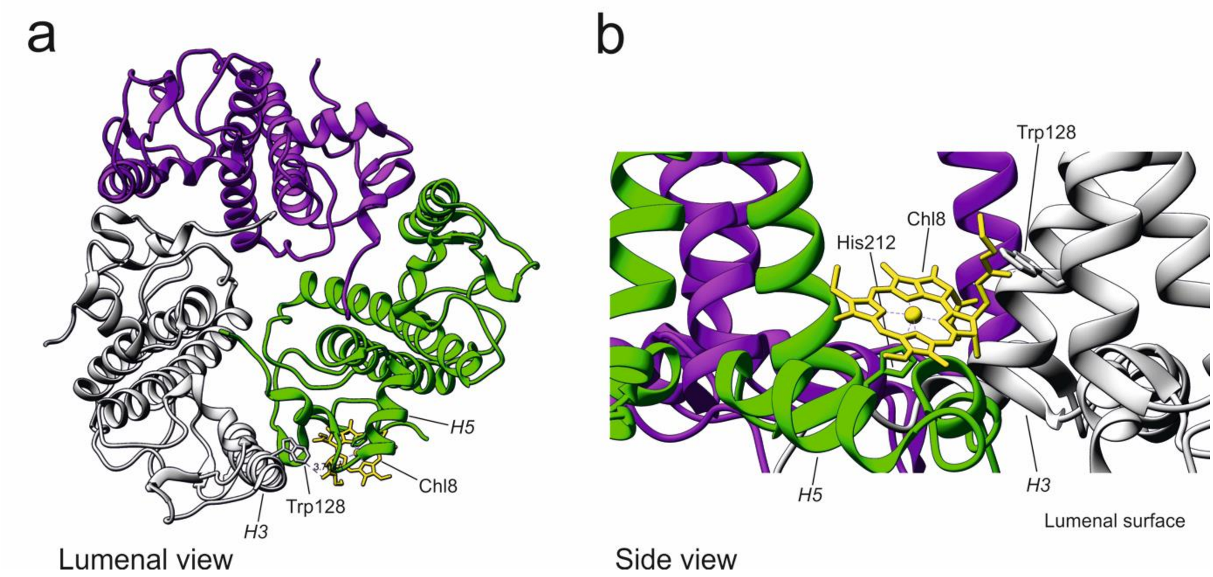


**Supplementary Figure 3** Chl8 mediated stabilization of trimeric LHCII between Trp128 and His212 of adjacent monomers. (**a**) Lumenal view and (**b**) side view of the LHCII trimer with each monomer represented by a different color. Stabilization upon trimerization at peak 3 and 4 from **Fig. 2b (top)** is facilitated as the central $Mg^{2+}$ of Chl8 (yellow) is coordinated at His212 (*lc* = 77 nm) of one monomer (green), while the hydrophobic phytol tail of Chl8 interacts with Trp128 (*lc* = 47 nm) of an adjacent monomer (gray); structure obtained with Chimera and PDB ID code 2BHW.
